# Supplementary material for: MetaRibo-Seq measures translation in microbiomes
Source: Nat Commun. 2020 Jun 29;11:3268. doi: 10.1038/s41467-020-17081-z (PMC7324362; doi:10.1038/s41467-020-17081-z)
Supplement: Supplementary file 10 — Supplementary Data 7 [file 41467_2020_17081_MOESM10_ESM.zip › File2/Confidence_High_Taxonomy/311046_out.krona.html]

Javascript must be enabled to view this page.

members
magnitude
magnitudeUnassigned
count
unassigned
taxon
rank

311046\_out

3

superkingdom
2
2

2
1239
phylum

class
2
186801

2
186802
order

1
1715004

SRS056273\_contig\_number\_14800
species

family
541000
1

genus
1263
1

1638786

SRS020233\_contig\_number\_contig-100\_21773.53765
1
species

2759
1
superkingdom

kingdom
1
4751

subkingdom
451864
1

1
4890
phylum

1
147538
subphylum

1
147548
class

1
5178
order

family
1
28983

genus
2082975
1

2259768

SRS104400\_contig\_number\_447
1
species
